# Assessment of short-term prognosis by sinus heart rate turbulence in patients with unstable angina

**DOI:** 10.3892/etm.2013.953

**Published:** 2013-02-08

**Authors:** ZHEN-QIANG SHENG, YE-FEI LI, GANG LIN, YI WANG, HUI-HE LU

**Affiliations:** Department of Cardiology, The Second Affiliated Hospital of Nantong University, Nantong 226001, P.R. China

**Keywords:** unstable angina, predictor, sinus heart rate turbulence

## Abstract

The aim of this study was to explore the correlation between sinus heart rate turbulence (HRT) and short-term prognosis in patients with unstable angina (UA). Seventy-five patients with UA received Holter monitoring for 24 h, within 48 h of hospitalization to obtain parameters of HRT, including turbulence onset (TO) and turbulence slope (TS), as well as parameters of heart rate variability (HRV), including standard deviation of all NN intervals (SDNN) and average R-R interval. The left ventricular ejection fraction (LVEF) was measured with an ultrasound cardiogram. Patients were divided into a stable group and a refractory group based on the prognosis during a 7- to 21-day hospital stay. The correlation between the prognosis and each risk factor was analyzed. Of the 75 patients with UA, the pathogenetic condition was stable in 50 patients (stable group) and cardiac events occurred in 25 patients (refractory group). Univariate analysis indicated that the risk factors of short-term poor prognosis of UA include TS ≤2.5 msec/R-R, age ≥70 years, LVEF <40% and SDNN <70 msec. Logistic multivariate regression analysis revealed that only TS ≤2.5 msec/R-R and LVEF <40% were independent risk factors of short-term poor prognosis. Our study revealed that weakening or disappearance of HRT is an independent predictor of short-term poor prognosis in patients with UA.

## Introduction

The condition of unstable angina (UA) changes quickly and easily develops into acute myocardial infarction (AMI) in a number of patients. It is important to identify high-risk UA patients early and adopt active treatment. Therefore, it is necessary to identify a new method to predict high-risk UA patients. At present, the methods to predict the risk of acute coronary syndrome include assessment of clinical symptoms, signs, electrocardiogram (ECG), biochemical markers and imaging techniques, including myocardial perfusion imaging, cardiovascular magnetic resonance imaging and computed tomography (CT) coronary angiography. These methods have their advantages and disadvantages. The diagnostic sensitivity and specificity of imaging technology is acceptable; however, it is time-consuming and its cost is high, which is not conducive to repetitious examinations (1). In patients with UA, the biochemical marker troponin may not be elevated and brain natriuretic peptide (BNP) and C-reactive protein (CRP) have poor specificity (2). Although electrocardiography is simple, it is difficult to use it to predict AMI risk in patients with arrhythmia (3,4). Previously, it was reported that sinus heart rate turbulence (HRT) has good predictive value in patients with myocardial infarction (5). However, little research has been performed with regard to whether HRT is able to predict the prognosis in patients with UA. The purpose of this study was to evaluate the predictive value of HRT for the prognosis of patients with UA.

## Materials and methods

### Subjects

Seventy-five patients were diagnosed with UA by coronary angiography according to the American College of Cardiology/American Heart Association (ACC/AHA) diagnosis and treatment guidelines in 2007 (6). Patients were divided into a stable group (50 patients) and a refractory group (25 patients) based on the prognosis during the 7- to 21-day hospital stay. The refractory group was defined as the patients having one of the following events following drug treatment, including aspirin, clopidogrel, low molecular weight heparin (LMWH), statin, beta-receptor blocker, calcium channel blocker and nitrates: i) resting angina; ii) no improvement in symptoms and exercise tolerance with the requirement of percutaneous coronary intervention (PCI) or coronary artery bypass grafting (CABG) and iii) AMI or cardiac mortality. The stable group was defined as the patients presenting clear improvement in symptoms and exercise tolerance following drug treatment. The following patients were excluded from this study: i) patients with concomitant infection, tumor, hepatic or renal dysfunction; ii) patients with atrioventricular block and iii) patients taking anti-arrhythmic drugs for a long-term period. All study methods were approved by the ethics committee of the Second Affiliated Hospital of Nantong University. All the subjects enrolled into the study provided written formal consent to participate.

### Methods

Patients underwent 24-h Holter monitoring and echocardiography within 48 h of hospitalization. Following Holter ECG recordings, the Holter file was analyzed with Pathfinder 700 analyzer system (Reynolds Medical Ltd., Hertford, UK) to obtain the values of turbulence onset (TO) and turbulence slope (TS). The TO value was calculated according to the following formula: TO = (mean of the first two sinus rhythm R-R intervals after the ventricular compensatory interval - mean of the two sinus rhythm R-R intervals before the cycle of ectopic beat) / mean of the two sinus rhythm R-R intervals before the cycle of ectopic beat. For the TS value, the first 20 ventricular rhythm R-R intervals after premature ventricular beats were measured and then a scattergram of the R-R interval was created with the serial number of the R-R intervals as the x-axis and with the values of R-R intervals as the y-axis. The sinus rhythm R-R intervals of 5 consecutive serial numbers were randomly selected to create a regression line and then the positive maximum slope served as the TS values. TO values were expressed as percentages and TS as msec/R-R. Additionally, the Holter monitor also produced the heart rate variability (HRV) and standard deviation of all NN intervals (SDNN). The left ventricular ejection fraction (LVEF) was measured with an ultrasound cardiogram (Philips iE33 System, Netherlands). The cut-off points for each risk factor (7–10) are shown in Table I.

### Statistical analysis

Statistical analysis was performed with SPSS 11.0 software (SPSS Inc., Chicago, IL, USA). Independent sample t-test or rank sum test were used in the comparisons of measurement data. The Chi-square test was used in the comparisons of enumeration data. Logistic multivariate regression analysis was performed with poor prognosis as dependent variables and with other parameters as independent variables. P<0.05 was considered to indicate a statistically significant difference.

## Results

### General status

The pathogenetic condition of the 50 patients in the stable group was improved. In the 25 patients of the refractory group, 18 had intractable angina requiring revascularization (15 received PCI and 3 CABG), 5 had AMI and 2 succumbed of ventricular arrhythmia. The general information in the two groups is shown in Table II. With the exception of TO, TS, LVEF and SDNN, there were no statistical differences in the parameters between the two groups.

### Logistic univariate and multivariate regression analyses of the risk factors for short-term poor prognosis

Logistic univariate regression analysis indicated that the risk factors of short-term poor prognosis were TS ≤2.5 msec/R-R, age ≥70 years, LVEF <40% and SDNN <70 msec. Logistic multivariate regression analysis revealed that the risk factors of short-term poor prognosis were TS ≤2.5 msec/R-R and LVEF ≤40% with independent predictive value (Table III).

## Discussion

The risk predictors of acute coronary syndrome include serum biomarkers (troponin I, BNP and CRP), ECG analysis and imaging techniques. The diagnostic sensitivity and specificity of imaging technology is acceptable; however, it is time-consuming and its cost is high, which is not conducive to repetitious examinations (1). BNP has poor specificity (2) and the predictive value of CRP is less in patients with UA than in patients with ST segment elevation myocardial infarction (STEMI) or non-STEMI (11). Often, conventional detection does not reveal the increased troponin I in the patients with UA. Although the high-sensitivity detection of troponin I has better predictive value, it is too expensive to use widely in clinical practice (12). The predictive values of new markers are limited (13,14). The predictive markers for UA are fewer. Therefore, it is important to find a simple and reliable predictor for UA. HRT is a new high-risk predictor that has good predictive value for cardiac events in patients with AMI (15,16). However, little research has been performed to investigate whether HRT is able to predict short-term poor prognosis in patients with UA.

In the present study, weakening or disappearance of HRT was a risk factor of short-term poor prognosis in the refractory group and has independent predictive value in patients with UA. The weakening or disappearance of HRT as a predictor of short-term poor prognosis is closely related to the HRT formation mechanism. In HRT, the sinus rhythm first accelerates and then decreases following a premature ventricular beat with a compensatory pause. The integrity and stability of the autonomic nervous system are evaluated according to the changes in ECG rhythm caused by a premature ventricular beat. HRT is caused by the direct effects of premature ventricular beats and baroreflex. There are sympathetic and parasympathetic nerves and adrenergic and muscarinic receptors in epicardial and coronary arteries. Acute myocardial ischemia allows receptor terminals to be damaged, the impulses from the sympathetic and vagus nerves to be abnormal and the equilibrium state of the autonomic nervous system to be destroyed, leading to the weakening or disappearance of HRT in patients with ischemic heart disease (17–19).

Lin *et al* (20,21) reported that the vagus nerve plays an important role in HRT. Vagus tone has certain anti-arrhythmic effects. In patients with ischemic heart disease, due to autonomic nervous imbalance and vagus nerve dysfunction, the anti-arrhythmic effects are lost and the sudden mortality rate is increased with an elevated TO value and decreased TS value.

Bonnemeier *et al* (22) identified that once AMI is treated with PCI, the TS value increases and TO value decreases in patients with thrombolysis in myocardial infarction (TIMI) flow grade 3; however, in patients with TIMI flow grade 2, the values of TS and TO were not markedly changed, demonstrating that successful reperfusion following PCI improves HRT and allows the rapid recovery of the baroreceptor reflex. Abnormal HRT may be used as a valuable new indicator of severe myocardial ischemia.

In this study, logistic univariate and multivariate regression analyses were performed for the risk factors of short-term poor prognosis in patients with UA, including age, HRT, LVEF, SDNN and ST segment displacement. Our results indicate that only TS ≤2.5 msec/R-R and LVEF ≤40% have independent predictive value. Weakening and disappearance of HRT following premature ventricular beats may serve as a risk indicator for patients with UA.

The limitations of this study include a small sample size, no stratified analysis for subgroups and no comparison of prognostic values between HRT and biomarkers.

## Figures and Tables

**Table I:** Cut-off points for each risk factor.

Risk degree	Age (years)	OMI history	SDNN (msec)	TO (%)	TS (msec/R-R)	LVEF (%)	ST segment displacement (mm)	R-R interval (msec)
High risk	≥70	Yes	<70	≥0	≤2.5	≤40	≥1	<800
Low risk	<70	No	≥70	<0	>2.5	>40	<1	≥800

OMI, old myocardial infarction; SDNN, standard deviation of all NN intervals; TO, heart rate turbulence onset; TS, heart rate turbulence slope; LVEF, left ventricular ejection fraction.

**Table II:** General information on the stable and refractory groups.

Parameters	Stable group (n=50)	Refractory group (n=25)	P-value
Males	32 (64%)	18 (72)	0.064
Age (years)	64.3±7.2	63.1±8.3	0.102
Smoking	19 (38)	11 (44)	0.053
Hyperlipidemia	20 (40)	9 (36)	0.12
Diabetes	9 (18)	5 (20)	0.065
Hypertension	28 (56)	15 (60)	0.077
BMI (kg/m^2^)	25.1±2.7	25.4±3.1	0.68
OMI	6 (12%)	4 (16)	0.053
Drug use			
Aspirin	48 (96)	23 (92)	0.35
Clopidogrel	48 (96)	23 (92)	0.35
LMWH	47 (94)	23 (92)	0.43
Statin	49 (98)	25 (100)	0.46
Beta-receptor blocker	45 (90)	21 (84)	0.23
Calcium channel blocker	37 (74)	19 (76)	0.52
Nitrates	47 (94)	24 (96)	0.44
TO (%)	−0.59±2.13	0.65±1.29^a^	0.0025
TS (msec/RR)	5.79±4.56	2.31±2.06^a^	0.0011
LVEF	59±12	46±15^a^	0.0001
SDNN (msec)	88.26±26.21	66.23±29.56^a^	0.0016

a P<0.05, compared with the stable group. Data are presented as n (%) or mean ± standard deviation. OMI, old myocardial infarction; BMI, body mass index; LMWH, low molecular weight heparin; TO, heart rate turbulence onset; TS, heart rate turbulence slope; LVEF, left ventricular ejection fraction; SDNN, standard deviation of all NN intervals.

**Table III:** Logistic regression analyses of the risk factors for short-term poor prognosis.

Variables	Univariate analysis	P-value	Multivariate analysis	P-value
Age ≥70 years	1.21 (0.96–1.97)	0.02	1.26 (1.04–1.93)	0.31
OMI history	1.25 (0.86–2.01)	0.07	1.24 (0.97–1.96)	0.26
SDNN <70 msec	1.22 (1.02–1.86)	0.02	1.19 (0.87–1.89)	0.18
TO ≥0	1.26 (1.09–2.11)	0.12	1.43 (1.13–2.78)	0.27
TS ≤2.5 msec/R-R	1.35 (1.07–1.99)	0.03	1.26 (0.94–2.76)	0.02
LVEF <40 %	1.09 (0.89–1.86)	0.01	1.31 (1.05–2.03)	0.01
ST segment displacement ≥1 mm	1.19 (0.87–1.68)	0.26	1.06 (0.79–1.68)	0.09
RR interval <800 msec	1.13 (0.94–1.57)	0.17	1.46 (1.13–2.94)	0.07

Data are presented as OR (95% CI). OR, odds ratio; CI, confidence interval; OMI, old myocardial infarction; SDNN, standard deviation of all NN intervals; TO, heart rate turbulence onset; TS, heart rate turbulence slope; LVEF, left ventricular ejection fraction.
